# Protocol of the Inner Mongolian Healthy Aging Study (IMAGINS): a longitudinal cohort study

**DOI:** 10.1186/s12889-022-12542-0

**Published:** 2022-01-17

**Authors:** Yunfeng Xi, Qiuyue Tian, Buqi Na, Ke Han, Mingrui Duan, Xingguang Zhang, Wenrui Wang, Youxin Wang

**Affiliations:** 1 The Inner Mongolia Autonomous Region Comprehensive Center or Disease Control and Prevention, Hohhot, 010000 China; 2 Beijing Key Laboratory of Clinical Epidemiology, School of Public Health, Capital Medical University, 10 YouanmenXitoutiao, Beijing, 100069 China; 3 School of Public Health, Inner Mongolia Medical University, Hohhot, 010000 China

**Keywords:** Cohort study, Inner Mongolia, Cardiovascular diseases, High-risk population

## Abstract

**Background:**

Cardiovascular diseases (CVDs) remain the leading cause of premature mortality and burden of diseases in the world. The Inner Mongolia Autonomous Region is located in northern China, constitute 17.66% individuals with Mongolian, which have unique diet and lifestyles. Therefore, the Inner Mongolian Healthy Aging Study (IMAGINS) was designed to explore risk factors for chronic diseases and evaluate the effectiveness of health management on CVDs in population at high-risk.

**Methods:**

The IMAGINS is an ongoing and prospective cohort study of men and women aged ≥35 years from Inner Mongolian Autonomous Region, northern China. This study performed in investigating risk factors for CVDs, screening and providing health management strategy for high-risk population of CVDs. The IMAGINS began in September 2015 and scheduled to recruiting and follow-up outcome until 2030. For general population, a long-term follow-up will be conducted every 5 years to collect the information above and data on clinical outcomes. For high-risk population, comprehensive health managements were performed and scheduled to follow-up annually. All IMAGINS participants are followed for incident CVDs and death.

**Discussion:**

The IMAGINS is designed to increase understanding how cardiovascular-related risk factors contribute to the development of CVDs and the positive effect of health management strategy for high-risk CVD participants. Key features of this study include (i) a carefully characterized cohort between high risk of CVDs and non-high risk population; (ii) detailed measurement of CVDs risk factors and health management strategies for high risk population; (iii) long-term follow-up of CVDs and death. The IMAGINS represents a good research opportunity to investigate clinical and genetic factors in high-risk population, might providing basis for the prevention and control of non-communicable diseases.

## Background

Cardiovascular diseases (CVDs) remain the leading cause of premature mortality and burden of diseases in the world [1, 2]. The number of total CVDs doubled from 271 million in 1990 to 523 million in 2019 worldwide [3]. The number of deaths from CVDs has risen substantially worldwide and reached 18.6 million in 2019 [3, 4]. In China, CVDs remains the top cause of death, which caused almost 4 million deaths in 2016 [5]. The number of CVDs (myocardial infarction [MI] and stroke) estimate to be 21 million, attributing from a multiplicity of cardiometabolic, behavioral, environmental, and social risk factors [6–9]. Therefore, CVDs still represent a great burden on healthcare systems worldwide, resulting in serious public health problems.

The Inner Mongolia Autonomous Region located in northern China, which has longest longitude and the second largest latitude in China. The unique geographical feature results in its climatic characteristics, such as, cold climate and temperature difference between east and west region [10]. A study revealed a positive association between temperature and stroke in Inner Mongolia [10]. The Inner Mongolia Autonomous Region is multi-ethnic, with Mongolian culture as the main body, constituted of 18,935,537 (78.74%) Han, 4,247,815 (17.66%) Mongolian, and 865,803 (3.60%) other ethnic groups [11, 12]. Most of Mongolian population lives as farmers and herders, and habitually engages high-fat, high-salt, whole milk, high rate of drinking alcohol, and less vegetable and fruit [13–15]. Due to unique climate, geography, ethnic culture and unhealthy lifestyles, the unique disease patterns were developed in Inner Mongolia differ from other regions [16]. In 2007, disability-adjusted life-years owing to CVDs in Inner Mongolia were significantly higher than other regions [17]. Previous studies showed that the prevalence of CVDs, hypertension and CVD mortality in the Inner Mongolia was higher than in the other regions, possibly attributing to difference of lifestyle and environment factors [18–22]. Hence, it’s necessary to perform a cohort study that investigate risk factors of CVDs or chronic diseases and screen high-risk CVDs population, according to difference in ethnic, diet, lifestyle, and geography in Inner Mongolia.

These cohort studies provided support for investigating association between risk factors and CVDs or chronic diseases, such as Prediction for Atherosclerotic CVD Risk in China (China-PAR) [23], Kailuan Study at Tangshan, Heibei [24], China Kadoorie Biobank (CKB) [25], and China suboptimal health cohort study (COACS) [26]. At present, there was only one cohort study for Mongolian population, which conducted among 2500 Mongolian population aged ≥20 years and investigated the risk factors of chronic diseases [27]. However, above cohort studies have not yet been focused on health management of high-risk CVDs population and unique risk factors of CVDs for Mongolian population. A common phenomenon, although highly prevalent CVDs and risk factors, is a very low proportion of older adults in China reported taking CVDs medications [28]. Studies showed that two-third of participants have hypertension, but only a third were being treated, even if treatment was considered beneficial [29, 30]. Similarly, a systematic review including 5 randomization trials demonstrated that general health checks did not decrease the incidence of CVDs, which the reason might be related to uncontrolled risk factors, poor self-health awareness or behavior, especially for high-risk CVDs population [31]. In addition, the prevalence of stroke and hypertension in the Mongolian population was higher than in the Han population, attributing from its unique climate, geographic location, and culture [20, 21]. Previous studies also reported that the awareness, treatment, and control rate of hypertension, diabetes, and CVDs in Inner Mongolia were low [16, 21, 32, 33]. Therefore, it is necessary that explored effectively health management strategies for high-risk CVDs population in Inner Mongolia, including primary and secondary CVDs prevention.

To this end, we started the Inner Mongolian Healthy Aging Study (IMAGINS), based on the China Patient-centered Evaluative Assessment of Cardiac Events (China-PEACE) Million Persons Project [34], a community-based longitudinal cohort. The major objectives of IMAGINS are to investigate factors for chronic disease, screen high-risk population of CVDs, and provide health management strategies for high-risk population. The rationale, design, methods and baseline characteristics of IMAGINS were described.

## Methods

### Study design and participants

**Fig. 1:**
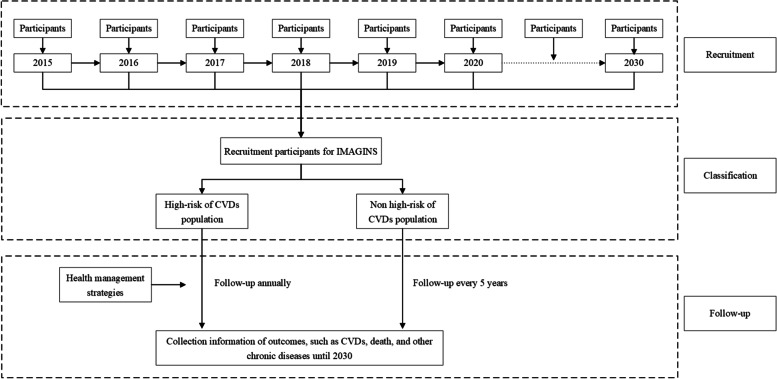
Study design of the IMAGINS. CVDs, cardiovascular diseases; IMAGINS, Inner Mongolian Healthy Aging Study

The IMAGINS is an open and community-based longitudinal study in Inner Mongolian Autonomous Region, China. This study performed in investigating risk factors for CVDs, screening and providing health management strategy for high-risk population of CVDs. The IMAGINS began in September 2015 and has continued into 2020. Recruitment and a long-term yearly follow-up will be performed until 2030, with the purposed of better understanding how cardiovascular-related risk factors contribute to the development of CVDs and the positive effect of health management strategy for high-risk CVD participants. The detail information was described in Fig. 1. The multistage stratified cluster sampling method was used to recruit study sample. The study selected randomly six cities (Hohhot, Erdos, Wuhai, Xingan league, Chifeng, and Hulun Buir) in Inner Mongolian Autonomous Region, based on geographic location, economic and demographic characteristics. In each city, one district or country was selected according to population stability and size of district. Then, in each district or country, 2-3 urban residential communities or rural villages were chosen based on size of communities or rural villages, population stability, and ability of local workers to perform the screening [33]. In each communities or villages, potential eligible participants were recruited by local workers via extensive publicity campaigns on the television and in the newspapers [33]. Participants aged ≥35 years old and living in Inner Mongolia Autonomous Region at least 6 months of the previous 1 year were recruited. Participants having history of CVDs (including self-reported MI, stroke, and coronary heart disease [CHD]) were excluded.

All the participants were invited to complete a questionnaire and physical examinations assessing cardiovascular-related health status for identifying high-risk CVD participants. For high-risk CVD participants, electrocardiograph, ultrasound scans, laboratory test, and extended questionnaire were tested for further assessment cardiovascular health status. The detail information was presented in Table 1. In addition, targeted personalized health management strategies were performed for high-risk population, including lifestyle intervention recommendations, primary prevention of CVDs, and secondary prevention of CVDs.

**Table 1:** Testing program in the Inner Mongolian Healthy Aging Study

Test	Baseline	Follow-up	
		Non high-risk	High-risk
Physical examination	Height, weight, BP, and WC	Height, weight, BP, and WC	Height, weight, BP, and WC
Physiological and biochemical indexes	**Blood biochemistry:** Blood glucose TC TG HDL-C LDL-C **Routine urine:** Urine protein Glucose Urine occult blood Acetone body	**Blood biochemistry:** Blood glucose TC TG HDL-C LDL-C **Routine urine:** Urine protein Glucose Urine occult blood Acetone body	**Blood biochemistry:** Blood glucose TC TG HDL-C LDL-C Apolipoprotein ALT AST Creatinine Urea nitrogen HbAlc CK CK-MB CRP **Routine urine:** Urine protein Glucose Urine occult blood Acetone body
Other examination	–	–	ECG and carotid artery ultrasound
Questionnaires	Baseline questionnaire	Follow-up questionnaire	Follow-up questionnaire Intervention records

*ALT* alanine aminotransferase, *AST* aspartate transaminase, *BP* blood pressure, *CK* creatine kinase, *CK-MB* creatine kinase isoenzyme MB, *CRP* high-sensitivity C-reactive protein, *ECG* electrocardiograph, *HbAlc* glycosylated hemoglobin, *HDL-C* high density lipoprotein cholesterol, *LDL-C* low density lipoprotein cholesterol, *TC* total cholesterol, *TG* triglyceride, *WC* waist circumference

Based on the sample size formula of cohort study, the cumulative incidence of cardiovascular events was reported to be 7.86% in 4 years with low cardiovascular risk population (non-exposed group) and 10.74% in 7 years with high cardiovascular risk population (exposed group) [35, 36], α = 0.5, β = 0.10, the prevalence ratio of high-risk CVD group (exposed group) vs. non-high-risk group (non-exposed group) of 1:4, the sample sizes of high-risk and non-high-risk CVD groups were estimated to be 2133 and 8532 respectively (PASS 11). Considering attrition rate of 10%, a sample size of 2346 for high-risk group and 9385 for non-high-risk group met the minimum required sample size. In actually, 140,000 participants were recruited, which is 11 times that of the required sample size.

This study was approved from ethics committee of the Inner Mongolia Autonomous Region Comprehensive Center for Disease Control and Prevention (NO. NMCDCIRB2021003). Written informed consent has also been obtained from each of the participants.

### Data collection by questionnaires

The questionnaire collected information on demographics, lifestyle factors, medical history, and family history [33, 34, 37]. Demographics included age, gender, marital status (unmarried, married, or divorced, widowed, or separated), type of hukou (rural, or urban), nationality (Han, Mongolian, or other), education level (senior high school or below, or college or above), occupation, household income (≤ 50,000 Yuan/year, or > 50,000 Yuan/year), and medical insurance status (urban employment basic medical insurance, urban resident basic medical insurance, new rural cooperative medical scheme, or other). Information on tobacco smoking (frequency, type, amount), alcohol consumption (frequency, type, or volume), dietary intakes of 12 traditional foods (including rice, wheat, grain, meat, poultry, seafood, egg, vegetable, pickle, fruit, bean, and milk), and physical activity (frequency and type of activities in occupational, commuting, domestic, and leisure time) were collected [25, 37].

Medical histories on hypertension, diabetes, MI, stroke, angina, dyslipidemia, chronic obstructive pulmonary disease (COPD), and heart operations were collected by questionnaire, as well as medical history including age at diagnosis of diseases. Medication histories on hypertension, diabetes, dyslipidemia, and other CVDs during 2 weeks were collected. In addition, above medical histories were also investigated in immediate family members.

### Physical examination

Height, weight, and waist circumference were measured by standard anthropometric techniques. Body mass index (BMI) was calculated as weight in kilograms divided by height in meters squared. Based on the Chinese-specific criteria [38], participants were categorized into lean (BMI < 18.5 kg/m^2^), normal weight (18.5 kg/m^2^ ≤ BMI < 24 kg/m^2^), overweight (24 kg/m^2^ ≤ BMI < 28 kg/m^2^), and obesity (BMI ≥ 28 kg/m^2^). Blood pressure and heart ratio were measured by electronic sphygmomanometer arm (Omron HEM-7430; Omron Corporation, Kyoto, Japan). Three readings of systolic blood pressure (SBP), diastolic blood pressure (DBP), and heart ratio were taken to calculate the mean value as the final value.

Blood samples were collected from the antecubital vein of all participants in the morning under fasting conditions. Blood samples were stored in vacuum tubes containing ethylene diamine tetraacetic acid (EDTA) and coagulation tubes. Blood samples were measured using auto-analyzer (BeneCheck PD-G001-2, Taiwan, China and CardioChek PA Analyser, Polymer Technology Systems, Indianapolis, Indiana, USA) at laboratory of the Inner Mongolia Autonomous Region Comprehensive Center or Disease Control and Prevention. Fasting blood glucose (FBG), total cholesterol (TC), triglyceride (TG), high-density lipoproteins cholesterol (HDL-C), and low-density lipoproteins cholesterol (LDL-C) were assessed. After on-site processing and centrifugation, blood samples were stored for biospecimen banking (− 80 °C).

### Assessment of the high-risk population

According to the collected baseline information, system automatically identified high-risk population. The CVDs high-risk was defined as the presence of one following criteria [34], as following, 1) history of MI or stroke, percutaneous coronary intervention, or coronary artery bypass grafting; 2) high blood pressure (SBP ≥ 160 mmHg or DBP ≥ 100 mmHg), LDL-C ≥ 160 mg/dL (4.14 mmol/L), or HDL-C < 30 mg/dL (0.78 mmol/L); 3) based on WHO CVDs risk prediction charts [39], 10-year CVDs risk ≥20%.

### Follow-up and outcome assessment

The study participants will be followed up via face-to-face interviews up to December 31, 2030, or up to the occurrence of a final event as defined in the study, or occurrence of death. For general population, a long-term follow-up will be conducted every 5 years to collect the information above and data on clinical outcomes. For high-risk population, a long-term follow-up will be conducted annually.

Primary outcomes were new set CVD events and death. CVD events included stroke, MI, ischemic heart disease, heart failure, atrial fibrillation, angina, and cardiac death. Information of CVD events was obtained from medical physicians and medical insurance system. Death was assessed using family report, death certificates, and medical insurance system.

The secondary outcomes will include type 2 diabetes (T2D), hypertension, dyslipidemia, and other chronic diseases (cancer, COPD, and chronic kidney disease). T2D is defined as those who self-reported diabetes, those having history of taking anti-diabetic medicine in the past 2 weeks, or those FBG level ≥ 7.0 mmol/L (126 mg/dl) [33]. Hypertension is defined as those with average SBP ≥ 140 mmHg or DBP ≥ 90 mmHg, those who self-reported diabetes, or those having history of taking anti-hypertension medicine in the past 2 weeks [40]. Dyslipidemia is defined as those with TC > 5.7 mmol/L, TG > 1.7 mmol/L, HDL-C < 1.0 mmol/L, LDL-C > 3.4 mmol/L, or having history of taking anti-hypertension medicine in the past 2 weeks [41]. Diagnosis of above chronic diseases was collected by medical physicians and medical insurance system.

### Statistical analyses

Continuous variables were presented as mean (standard deviation) or median (inter-quartile range). Categorical variables were presented as number (percentage). Chi-square test was used for the comparisons of categorical variables. The differences between groups were tested by t test, analysis of variance (ANOVA), or rank-sum test for continuous variables. Two-sided *P* < 0.05 was considered statistically significant.

Longitudinal analysis towards demographics, risk factors, biomarkers, outcomes and their relationship will be performed through liner regression, logistic regression, generalized mixed models, survival analysis, cox proportional hazard models, and time-depending cox regression. These analyses will be focus on identification of factors that are associated with the onset and incidence of chronic diseases or mortality and on the evaluation effect of health management for high-risk population.

### Baseline characteristics

**Fig. 2:**
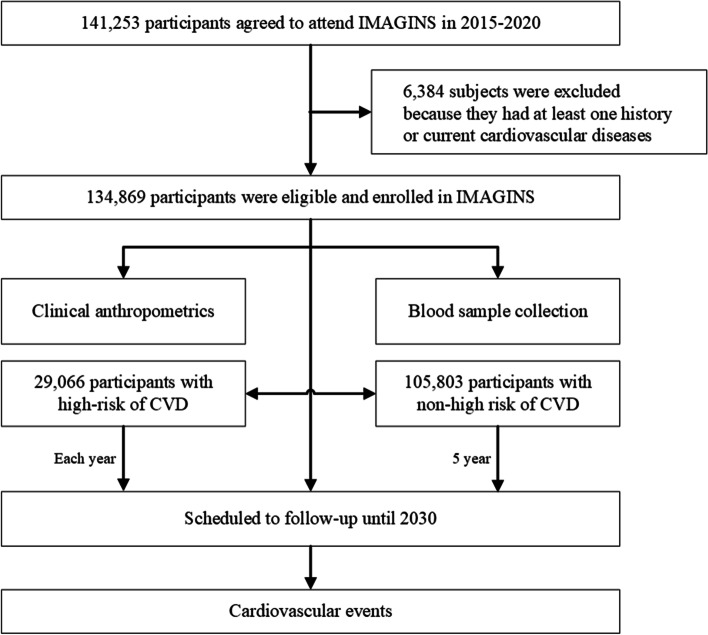
Flowchart of the Inner Mongolian Healthy Aging Study (IMAGINS). CVD, cardiovascular disease

At baseline, individuals without the occurrence of MI, stroke or CHD (*n* = 6384) were identified ensuring the cohort a sufficient sample size (Fig. 2). A total of 134,869 participants were enrolled in the IMAGINS until 2020, with 29,066 at higher risk of CVDs.

The baseline characteristics were summarized in Table 2. The median age of the participants at cohort entry was 54 years (IQR: 15) with 58.53% being women. The majority (79.05%) had a household income less than 50,000 Yuan per year, 90.10% were Han nationality, and 94.72% were married. About 10.69% of participants had completed college school or higher. Most of the participants reported never smoking (59.16%), never drinking (72.11%), had overweight (44.03%). The proportion of high-risk in the participants was 21.55%, higher in men than in women (22.96 vs. 20.56% in men and women, respectively). The age, gender, income, marital status, highest education completed, smoking status, drinking status, BMI, waist circumference, blood pressure, fasting blood glucose, TC, TG, HDL-C, LDL-C, and history of diseases (hypertension, diabetes, and dyslipidemia) differed significantly between the high-risk group and non-risk group (*P* < 0.05), whereas the differences of ethnicity was of no statistical significance (Table 3).

**Table 2:** Baseline demographic characteristics of the IMAGINS population stratified by gender^a^

Characteristics	Total (*N* = 134,869)	Men (*N* = 55,927)	Women (*N* = 78,942)	*P*
Age	54 (15)	55 (15)	54 (14)	< 0.0001
Nationality				0.0007
Han	121,512 (90.10)	50,587 (90.45)	70,925 (89.84)	
Mongolian	11,085 (8.22)	4452 (7.96)	6633 (8.40)	
Other	2272 (1.68)	888 (1.59)	1384 (1.75)	
Family Income				< 0.0001
< 50,000	99,254 (79.05)	40,319 (76.87)	58,935 (80.61)	
≥ 50,000	26,309 (20.95)	12,131 (23.13)	14,178 (19.39)	
Marital status				< 0.0001
Married with spouse	125,009 (94.72)	52,805 (96.23)	72,204 (93.65)	
Never married	615 (0.47)	556 (1.01)	59 (0.08)	
Widowed, separated, or divorced	6350 (4.81)	1511 (2.75)	4839 (6.28)	
Highest education completed				< 0.0001
High school	117,107 (89.31)	47,442 (86.93)	69,665 (91)	
College school or higher	14,023 (10.69)	7134 (13.07)	6889 (9)	
Smoking history				< 0.0001
Never	56,366 (59.16)	13,491 (29.17)	42,875 (87.44)	
Current	34,475 (36.18)	28,883 (62.45)	5592 (11.40)	
Former	4440 (4.66)	3875 (8.38)	565 (1.15)	
Drinking history				< 0.0001
Never	96,617 (72.11)	25,938 (46.77)	70,679 (90.01)	
Moderately	23,450 (17.50)	16,673 (30.07)	6777 (8.63)	
Heavy	13,913 (10.38)	12,843 (23.16)	1070 (1.36)	
Body mass index (kg/m^2^)				< 0.0001
< 18.5	1821 (1.35)	672 (1.20)	1149 (1.46)	
18.5-23.9	36,981 (27.42)	14,971 (26.77)	22,010 (27.88)	
24-27.9	59,389 (44.03)	24,686 (44.14)	34,703 (43.96)	
≥ 28	36,678 (27.20)	15,598 (27.89)	21,080 (26.70)	
Waist circumference (cm)	85 (13)	89 (14)	83 (13)	< 0.0001
Blood pressure (mmHg)				
Systolic blood pressure	136 (28)	136 (25)	136 (29)	< 0.0001
Diastolic blood pressure	83 (15)	85 (15)	82 (16)	< 0.0001
Fasting blood glucose (mmol/L)	6 (1)	6 (2)	6 (1)	< 0.0001
Total cholesterol (mmol/L)	4 (1)	4 (1)	5 (1)	< 0.0001
Triglycerides (mmol/L)	1 (1)	1 (1)	1 (1)	< 0.0001
High-density lipid cholesterol (mmol/L)	1 (1)	1 (1)	1 (1)	< 0.0001
Low-density lipid cholesterol (mmol/L)	2 (1)	2 (1)	2 (1)	< 0.0001
History of hypertension	71,267 (52.84)	30,755 (54.99)	40,512 (51.32)	< 0.0001
History of dyslipidemia	11,453 (8.49)	3415 (6.11)	8038 (10.18)	< 0.0001
History of diabetes	23,016 (17.07)	10,473 (18.73)	12,453 (15.89)	< 0.0001
CVD High-risk	29,066 (21.55)	12,839 (22.96)	16,227 (20.56)	< 0.0001

*CVD* cardiovascular disease, *IMAGINS* Inner Mongolian Healthy Aging Study

^a^Continuous variables were represented as median (interquartile range), while discrete variables were represented as number (proportion)

**Table 3:** Factors distribution in participants with or without high-risk of CVDs^a^

Characteristics	Total (N = 134,869)	High-risk (*N* = 29,066)	Non-high risk (*N* = 105,803)	*P*
Age	54 (15)	57 (14)	54 (14)	< 0.0001
Gender				< 0.0001
Men	55,927 (41.47)	12,839 (44.17)	43,088 (40.72)	
Women	78,942 (58.53)	16,227 (55.83)	62,715 (59.28)	
Nationality				0.2077
Han	121,512 (90.10)	26,183 (90.08)	95,329 (90.10)	
Mongolian	11,085 (8.22)	2361 (8.12)	8724 (8.25)	
Other	2272 (1.68)	522 (1.80)	1750 (1.65)	
Household income per year				< 0.0001
< 50,000	99,254 (79.05)	21,469 (80.44)	77,785 (78.67)	
≥ 50,000	26,309 (20.95)	5220 (19.56)	21,089 (21.33)	
Marital status				< 0.0001
Married with spouse	125,009 (94.72)	26,410 (93.82)	98,599 (94.97)	
Widowed, separated, or divorced	615 (0.47)	118 (0.42)	497 (0.48)	
Never married	6350 (4.81)	1623 (5.77)	4727 (4.55)	
Highest education completed				0.0002
High school	117,107 (89.31)	25,123 (89.93)	91,984 (89.14)	
College school or higher	14,023 (10.69)	2814 (10.07)	11,209 (10.86)	
Smoking history				< 0.0001
Never	56,366 (59.16)	10,834 (55.61)	45,532 (60.07)	
Current	34,475 (36.18)	7576 (38.89)	26,899 (35.49)	
Former	4440 (4.66)	1073 (5.51)	3367 (4.44)	
Drinking history				< 0.0001
Never	96,617 (72.11)	20,200 (70)	76,417 (72.69)	
Moderately	23,450 (17.50)	4891 (16.95)	18,559 (17.65)	
Heavy	13,913 (10.38)	3767 (13.05)	10,146 (9.65)	
Body mass index (kg/m^2^)				< 0.0001
< 18.5	1821 (1.35)	246 (0.85)	1575 (1.49)	
18.5-23.9	36,981 (27.42)	5301 (18.24)	31,680 (29.94)	
24-27.9	59,389 (44.03)	12,638 (43.48)	46,751 (44.19)	
≥ 28	36,678 (27.20)	10,881 (37.44)	25,797 (24.38)	
Waist circumference (cm)	85 (13)	88 (13)	84 (13)	< 0.0001
Blood pressure (mmHg)				
Systolic blood pressure	136 (28)	165 (19)	132 (20)	< 0.0001
Diastolic blood pressure	83 (15)	96 (16)	81 (14)	< 0.0001
Fasting blood glucose (mmol/L)	6 (1)	6 (1)	6 (1)	< 0.0001
Total cholesterol (mmol/L)	4 (1)	5 (2)	4 (1)	< 0.0001
Triglycerides (mmol/L)	1 (1)	2 (1)	1 (1)	< 0.0001
High-density lipid cholesterol (mmol/L)	1 (1)	1 (1)	1 (1)	< 0.0001
Low-density lipid cholesterol (mmol/L)	2 (1)	3 (1)	2 (1)	< 0.0001
History of hypertension	71,267 (52.84)	26,764 (92.08)	44,503 (42.06)	< 0.0001
History of diabetes	23,016 (17.07)	7357 (25.31)	15,659 (14.80)	< 0.0001
History of dyslipidemia	11,453 (8.49)	5592 (19.24)	5861 (5.54)	< 0.0001

*CVD* cardiovascular disease

^a^Continuous variables were represented as median (interquartile range), while discrete variables were represented as number (proportion)

## Discussion

The IMAGINS was designed to systematically and prospectively investigate these multiple factors affecting chronic diseases and evaluate the effect of health management for high-risk population, conducting research from disease prevention perspective in high-risk population. In IMAGINS, we defined the CVDs high-risk population using multiple indexes including blood pressures, history of CVDs or medicine, blood lipid, and risk assessment charts of CVDs. The high-risk group was considered as target to reduce CVDs risk, saving cost and time [42]. To our knowledge, this is the first cohort study that includes having Mongolian geographic and demographic characteristics and identification of high-risk CVDs population, which will enable to identify the high-risk indexes and provide personalized health management strategies.

At baseline of IMAGINS, high-risk participants differed from the non-high-risk participants with respect to age, gender, income, marital status, highest education completed, smoking status, drinking status, BMI, waist circumference, blood pressure, fasting blood glucose, TC, TG, HDL-C, LDL-C, and history of diseases. In addition, we did not observe the differences of ethnicity between high-risk and non-high-risk group. Although no significant difference between groups, ethnic specific genetic susceptibility for CVDs risk still need to be considered among Mongolian and Han adults [21, 43–45]. Women were over-represented (55.83%) in the high-risk group, indicating that women have higher risk of developing CVDs. Nature physiological difference or social environment factors may contribute to this phenomenon [46]. For high-risk group, almost 80% were low income (< 50,000 yuan/year of household income) and lower educational levels (high school or lower) participants. In addition, never smoking and never drinking appears to be protective factors for CVDs high-risk, suggesting that health lifestyles are associated with lower risk of CVDs [47, 48]. Furthermore, a prospective study including 2 US cohorts suggests that even if regularly taking prevention medicine, healthy lifestyle remains important [49]. Participants with obesity or overweight comprised the largest number of high-risk (80.92%) participants compared with other groups, which is known risk factor of CVDs or chronic diseases [50–53]. Additionally, in high-risk and non-high-risk group, diseases of hypertension were 92.08 and 42.06%, diseases of dyslipidemia were 19.24 and 5.54%, and diseases of diabetes was 25.31 and 14.80%, respectively. This study also revealed difference in the incidence of diseases among groups which indicated different CVDs risk.

Strengths of our study were discussed as follows. Firstly, we recruited permanent residents aged ≥35 years old as the study population in Inner Mongolia. To our knowledge, this is the first cohort study that includes health management strategies for high-risk CVDs population in Inner Mongolia, which will provide a new perspective from ethnic or region perspective for prevention of CVDs. Secondly, follow-up high-risk population will be conducted every year and data for effect of health management, risk factors and outcomes can be closely collected and updated. In addition, for non-high-risk population, we conduct follow-up every 5 years and further identify individuals who progress to CVDs high-risk, providing early health management strategies. Thirdly, approximately 140,000 participants were included at the prospective cohort study, ensuring sufficient statistical power and stability of the findings.

There are still several limitations to this study. Firstly, self-reported information from questionnaire may lead to recall bias. Repeated measurement of variables (questionnaire and blood biochemical indexes) will be evaluated by follow-up reports to minimize bias. Secondly, herders in Inner Mongolia might not be recruited because of a high degree of mobility of population. In addition, the IMAGINS recruited 140,000 participants in Inner Mongolia, accounting for 0.58% of the entire Inner Mongolia population, which might influence the representativeness of study sample. Thirdly, we conduct follow-up every 5 years for non-high-risk population, which might be missed follow-up information of important variables.

This study is a large cohort study to investigate risk factors related to onset and etiology of chronic diseases, perform deciphering the important relationships between disease process and risk factors within individuals, and further provide health management strategy for high-risk population in Inner Mongolia. Furthermore, the IMAGINS will provide a way for early screening, prevention and health management of CVDs.

## Data Availability

The datasets used or analyzed during the current study are available from the corresponding author on reasonable request.

## Abbreviations

ANOVA: Analysis of variance
BMI: Body mass index
CHD: Coronary heart disease
China-PAR: Prediction for Atherosclerotic CVD Risk in China
China-PEACE: China Patient-centered Evaluative Assessment of Cardiac Events
CKB: China Kadoorie Biobank
COACS: China suboptimal health cohort study
COPD: Chronic obstructive pulmonary disease
CVDs: Cardiovascular diseases
DBP: Diastolic blood pressure
EDTA: Ethylene diamine tetraacetic acid
FBG: Fasting blood glucose
HDL-C: High-density lipoproteins cholesterol
IMAGINS: Inner Mongolian Healthy Aging Study
LDL-C: Low-density lipoproteins cholesterol
MI: Myocardial infarction
SBP: Systolic blood pressure
TC: Total cholesterol
TG: Triglyceride
T2D: Type 2 diabetes
